# Congenital contractures and distinctive phenotypic features consistent with Stuve-Wiedmann syndrome in a male infant

**DOI:** 10.1186/1757-1626-1-121

**Published:** 2008-08-21

**Authors:** Ali Al Kaissi, Monika Rumpler, Robert Csepan, Franz Grill, Klaus Klaushofer

**Affiliations:** 1 Ludwig Boltzmann Institute of Osteology, at the Hanusch Hospital of WGKK and, AUVA Trauma Centre Meidling, 4th Medical Department, Hanusch Hospital, Vienna, Austria; 2 Orthopaedic Hospital of Speising, Paediatric Department, Vienna, Austria

## Abstract

**Introduction:**

Expressionless face associated with multiple contractures has been encountered in an infant. There is a wide range of misconception regarding the categorization of children with multiple contractures among different pediatric disciplines. The fundamental element in categorizing children with multiple contractures is "the etiological understanding". In the absence of concomitant neuromuscular disease, however, the search for other reasons is mandatory. Our present paper signifies the necessity of proper interpretations of unusual clinical and radiographic features.

**Case presentation:**

We describe a 3-months-old-infant presented with the phenotypic and the radiographic features consistent with the diagnosis of Stüve-Wiedemann syndrome. We report what might be the first clinical report of Stüve-Wiedemann syndrome from a consanguineous family in Austria.

**Conclusion:**

Congenital limitations of the hips in a newborn infant raise the possibility of " Congenital Hip Dislocation". As congenital hip dislocation is a dysplastic process. Here further knowledge by the pediatrician and the orthopaedic surgeon is needed. Our present patient appears to constitute a distinct pathological entity consistent with Stüve-Wiedemann syndrome (SWS). Superti-Furga et al, and Cormier-Daire et al, also suggest that Stüve-Wiedemann syndrome and Schwartz-Jampel syndrome type 2 are allelic conditions. We wish to stress that, given the rarity of syndromic malformation complex, our impression is that it is more common than it is reported.

## Introduction

Stuve-Wiedemann syndrome (SWS) is an autosomal recessive disorder characterized by bowing of the long bones and other skeletal anomalies, episodic hyperthermia, and respiratory and feeding distress usually resulting in early death [1,2]. Stüve-Wiedemann syndrome belongs to the group of congenital bowing disorders of bone, but corresponds to a specific condition different from camptomelic and kyphomelic dysplasias on the basis of distinctive radiographic manifestations, which include stubby long bones with internal cortical thickening and large metaphyses [3-6]. In 1998, SWS was merged with another rare skeletal dysplasia, Schwartz-Jample syndrome, type 2. Schwartz-Jampel syndrome (SJS) is a term applied to 2 different autosomal recessive inherited conditions, sometimes termed SJS type I and SJS type II. SJS type I has recognized subtypes, IA and IB, which are similar except that type IB manifests earlier and with greater severity. The most commonly recognized and described type is IA, which exhibits muscle stiffness, mild and largely nonpregressive muscle weakness, and a number of minor morphological abnormalities. In affected patients, problems with motor development frequently become evident during the first year of life. Usually, the characteristic dysmorphic features lead to an early diagnosis, no later than the age of 3 years. Types IB and type II now known to be a separate disease more commonly referred to as Stüve-Wiedmann syndrome [3-6]. SWS is phenotypically similar to SJS type IA and IB, but in practice we believe that SWS do manifests the abnormal features earlier and the prognosis is unpleasant. Furthermore, in Schwartz-Jampel syndrome type 1, it is true that it is phenotypically similar but genetically it is a distinct disorder caused by mutation in the HSPG2 gene on chromosome 1p36.1-p34. Parental consanguinity in our present patient is highly suggestive of autosomal recessive inheritance. We report what might be the first clinical report of SWS from a consanguineous family in Austria.

## Case presentation

The child was referred to the orthopaedic department by the pediatrician because of a suspicion of congenital hip dislocation! Referral was done at the age of 7 weeks. He was a product of normal gestation, at birth his growth parameters were around the 10^th ^percentile. The mother was a 25-years-old gravida 1 abortus 0 married to a 31-year-old related man (first cousin). At birth respiratory distress syndrome was the major concern.

**Figure 1:**
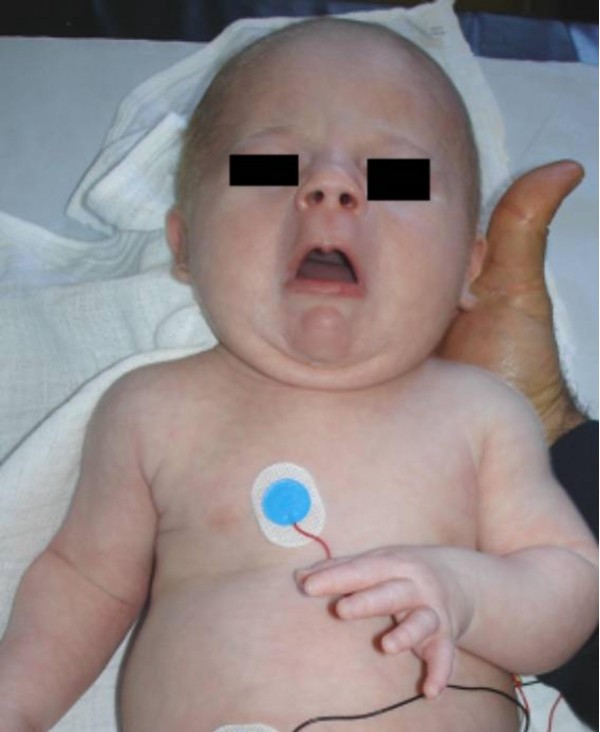
Proband's photo showed expressionless face associated with typical pursed appearance of the mouth, blepharophimosis, multiple contractures and umbilical hernia.

Examination showed growth around the 10 Th percentile. Expressionless face associated with typical pursed appearance of the mouth, blepharophimosis, multiple contractures and umbilical hernia (fig 1). The child had normal genetalia. Hearing, and vision were normal. All other investigations including an abdominal ultrasound, karyotyping, and metabolic tests, which aimed to test calcium, phosphorus, and vitamin D metabolism, were normal. Plasma carnitine and fatty acids, lactate/pyruvate ratios, and urinary organic acid excretions were assayed and found to be normal. Additional laboratory tests showed normal TSH/T4, a negative Guthrie test and normal karyotype. Search for specific mutations in the LIFR gene on chromosome 5p13 showed negative results as well.

**Figure 2:**
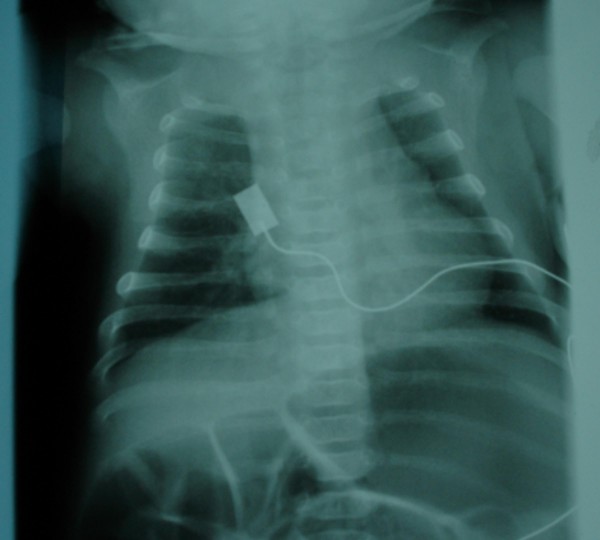
**Anteroposterior chest radiograph Chest radiogram showed a narrow upper thoracic cage (Bell-like) but with normal heart borders**.

**Figure 3:**
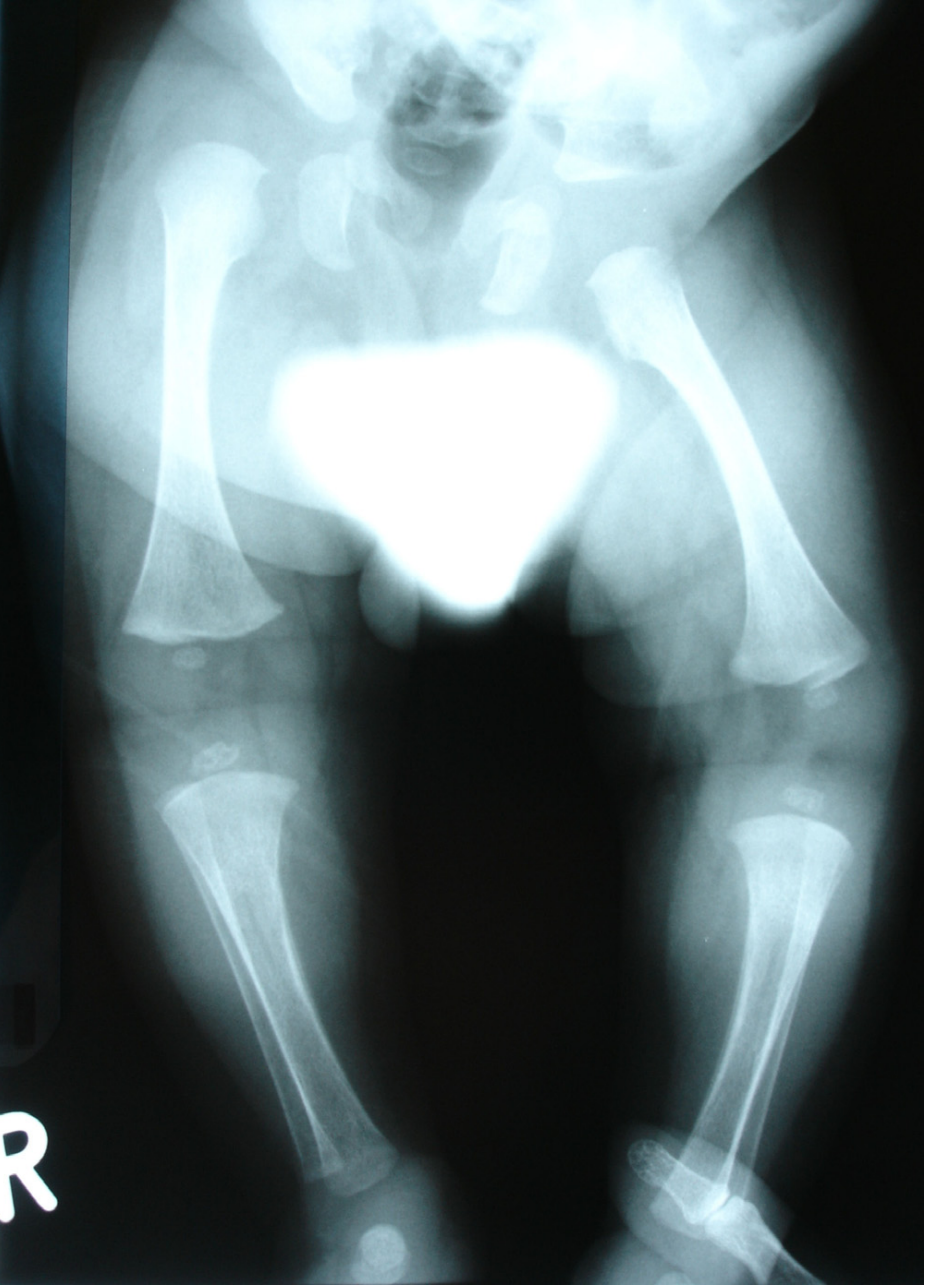
**Anteroposterior lower limb radiograph showed epiphyseal dysplasia of the capital femoral epiphyses, hypoplastic ileae, horizontally dysplastic acetabulae, coxa vara, shortening of the femoral necks, broad femora and tibiae with bilateral but asymmetrical degrees of mild bowing.** In addition the angulation of the femora was associated with internal thickening of the cortex.

Skeletal abnormalities include multiple joint contractures, thoracic kyphosis, mild bowing of the long bones, and a valgus deformity of the ankles. On the bases of skeletal survey, skull radiogram showed squared jaw with hypoplastic rami. Chest radiogram showed a narrow upper thoracic cage but with normal heart borders (fig 2). Spine radiograph showed platyspondyly and coronal clefts of the vertebral bodies. Lower limb radiograph showed epiphyseal dysplasia of the capital femoral epiphyses, hypoplastic ileae, horizontally dysplastic acetabulae, coxa vara, shortening of the femoral necks, broad femora and tibiae with bilateral but asymmetrical degrees of mild bowing. In addition the angulation of the femora was associated with internal thickening of the cortex. (fig 3). Upper limb radiograph showed joint contractures, associated with vertical lucencies in the metaphyseal region, mild bowing and thick cortices, and epiphyseal dysplasia with metaphyseal widening.

## Discussion

The clinical phenotype in our current patient plus the detailed radiographic documentation with absence of any biochemical marker in favor of a metabolic disorder was the baseline to establish the diagnosis of SWS. The cardinal features of Stüve-Wiedemann syndrome are joint contractures, bone dysplasia, and small stature. Severe respiratory difficulties and feeding problems are additional problems. Hypotonia rather than stiffness is prominent. Frequent bouts of hyperthermia have been described. A high infant mortality rate is a common association. Chen et al., [7] a case of a child surviving to age 9 years, stated that only 2 patients with long survival had been reported. In addition to problems with bone dysplasia, these children also manifest dysautonomic and neuropathic features, including reduced patellar reflex, lack of corneal reflex, and paradoxical perspiration at low temperatures [1-3].

Al-Gazali et al. [3] reported 3 children from 2 inbred Arab families with Stüve-Wiedemann syndrome who had survived the first year of life; their ages were 6, 2.8, and 2 years. All exhibited a characteristic phenotype resembling that described by Chen et al. [7] In all 3 children, the skeletal abnormalities progressed to severe bowing of the long bones with prominent joints and severe spinal deformity. All exhibited neurologic symptoms including temperature instability with excessive sweating, reduced pain sensation with repeated injury to the tongue and limbs, absent corneal reflexes, and a smooth tongue. There were also multiple fractures and progressive scoliosis. All 3 children had normal intelligence. Chabrol et al., [8] reported three cases from two consanguineous gypsy families. One case was noted to have hyperaminoaciduria and hepatic failure. Decreased activities of mitochondrial complex I and IV were found in two cases. There are also recurrent episodes of unexplained hyperthermia. Raas-Rothschild et al., [9] reported two sibs who died from severe pulmonary hypertension with pulmonary artery wall abnormality.

The cases of Stüve and Wiedemann [1,2] died in the neonatal period. Kozlowski and Tenconi's case [10] was aged three-and-a-half years and was said to have slight developmental delay. The differential diagnosis is camptomelic or kyphomelic dysplasia [11]. Siguady et al., [12] reported two fetuses with overlapping features between Stüve-Wiedemann syndrome and the neonatal form of Schwartz-Jampel syndrome. Cormier-Daire et al., [5] report six cases and provides a good review of the condition. Di Rocco et al., [13] reported a 13-year-old survivor (and a 3 year old case). They encountered the accumulations of lipid droplets in the muscles of their patients. Although what this means remains unclear and further research might elucidate the correlation. The gene has been mapped to 5p13 at locus D5S418 and mutations have been encountered in the leukemia inhibitory factor receptor (LIFR). Dagoneau et al [14] studied the genetic material of 19 patients who had been diagnosed with either SWS or SJS type II, they found that all patients had null mutations in their LIFR gene at the above-mentioned locus.

In summary, our current patient presented with congenital contractures, associated with temperature instability and excessive sweating. The latter seems to indicate a form of dysautonomia. Neither the pathophysiological mechanism nor the pathological course in our current patient seems similar to children with metabolic disorders. It is noteworthy to mention that cases of syndromic malformation complex are not uncommon for pediatricians/health care professionals; therefore, they should be appropriately informed on the subject. Their early identification/diagnosis requires adequate medical attention, since they will often be responsible for initial guidance that families receive.

## Abbreviations

SWS: Stüve-Wiedemann syndrome; SJS: Schwartz-Jampel syndrome; HSPG2 gene: heparan sulfate proteoglycan 2; LIFR: Leukemia inhibitory factor receptor alpha.

## Competing interests

The authors declare that they have no competing interests.

## Authors' contributions

All of the authors were involved in the clinico-radiographic assessment and finalising the paper. All authors have red and approved the final version of the paper.

## Consent

Written informed consent was obtained from the parents for the purpose of publication of the manuscript and figures of their child. A copy of the written consent is available for review by the editor-in-Chief of this journal.
